# Supplementary material for: Nephronectin-integrin α8 signaling is required for proper migration of periocular neural crest cells during chick corneal development
Source: eLife. 2022 Mar 3;11:e74307. doi: 10.7554/eLife.74307 (PMC8916771; doi:10.7554/eLife.74307)
Supplement: Supplementary file 1. [file elife-74307-supp1.docx]

**Supplementary File 1.** Table showing the primer sequences used for riboprobe synthesis.

| **Riboprobe Primer Sequences** | | | | |
| --- | --- | --- | --- | --- |
| **Name** | **Transcript** | **Forward (5'-3')** | **Reverse (5'-3')** | **Hyb Temp** |
| *Npnt* | XM_015276574 | CACCTCCAACGCCACCTCTA | AGCGTCCTCTTCTCAACATCACAT | 60 °C |
| *Itgα8* | XM_015281310 | CCACCTGAAGCAGATTACAC | ACCGCTAGTACCAGTAGACCA | 52 °C |
