# Supplementary material for: Nephronectin-integrin α8 signaling is required for proper migration of periocular neural crest cells during chick corneal development
Source: eLife. 2022 Mar 3;11:e74307. doi: 10.7554/eLife.74307 (PMC8916771; doi:10.7554/eLife.74307)
Supplement: Supplementary file 2. [file elife-74307-supp2.docx]

**Supplementary File 2.** Table showing the shRNA target sequences used for knockdown studies.

| **Chicken shRNA Sequences** | | |
| --- | --- | --- |
| **Label** | **Sequence** | **Location on Transcript** |
| Npnt-kd1 | GCTACTGTCTGAATGGCTACA | CDS493 |
| Npnt-kd2 | GGAGACAGTGCCAACCTATAT | CDS313 |
| Npnt-kd3 | GGAACAAACTGCATACAAAGG | CDS903 |
| Npnt-kd4 | GCTCGGAAAGTCTTAGTATTA | 3’UTR-341 |
| Itgα8-kd1 | GAGGAGACTCAGATGTAGACA | CDS1438 |
| Itgα8-kd2 | GCAGCATTTGAAGGCCAAAGT | CDS1647 |
| Itgα8-kd3 | GGGTTGAACGCAACAACAAGG | CDS2173 |
