# Supplementary material for: Nephronectin-integrin α8 signaling is required for proper migration of periocular neural crest cells during chick corneal development
Source: eLife. 2022 Mar 3;11:e74307. doi: 10.7554/eLife.74307 (PMC8916771; doi:10.7554/eLife.74307)
Supplement: Supplementary file 3. [file elife-74307-supp3.docx]

**Supplementary File 3.** Table showing the primers used to validate *Npnt* and *Itgα8* knockdown efficiency.

| **rt-PCR primers** | | | | |
| --- | --- | --- | --- | --- |
| **Name** | **Transcript** | **Forward (5'-3')** | **Reverse (5'-3')** | **Product size (bp)** |
| *Npnt* | XM_015276574 | CACCTCCAACGCCACCTCTACCTA | AGCGTCCTCTTCTCAACATCACAT | 708 |
| *Itgα8* | XM_015281310 | CCACCTGAAGCAGATTACAC | ACCGCTAGTACCAGTAGACCA | 1004 |
| *GAPDH* | NM_204305 | GATTCTACACACGGACACTTCA | CTGAGGGAGCTGAGATGATAAC | 229 |
