# Supplementary figures and images for: Nephronectin-integrin α8 signaling is required for proper migration of periocular neural crest cells during chick corneal development
Source: eLife. 2022 Mar 3;11:e74307. doi: 10.7554/eLife.74307 (PMC8916771; doi:10.7554/eLife.74307)

Figure 2-figure supplement 1C Source Data

**Npnt-kd**

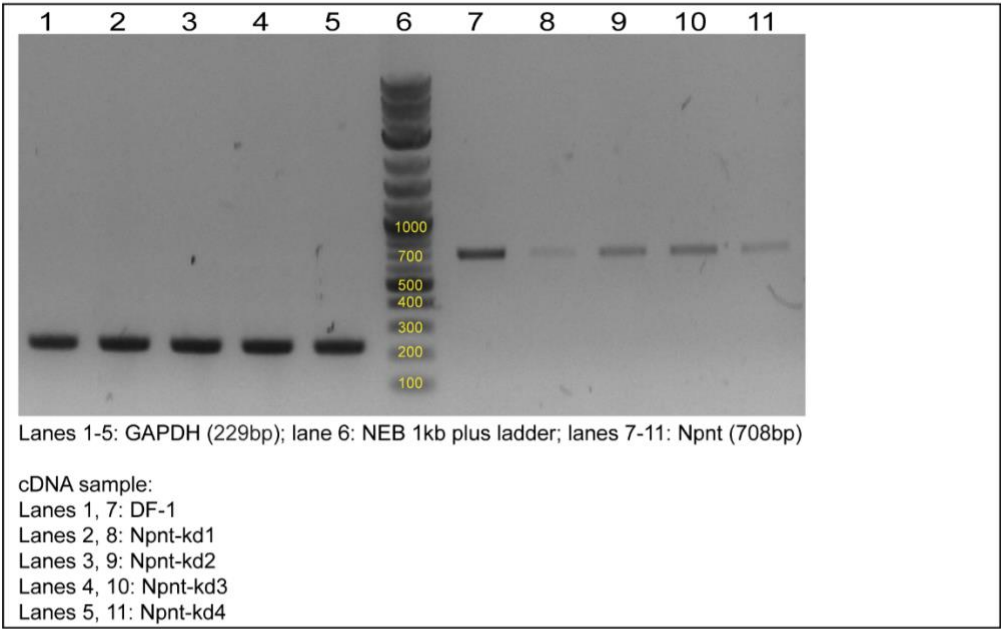

**Itga8-kd**

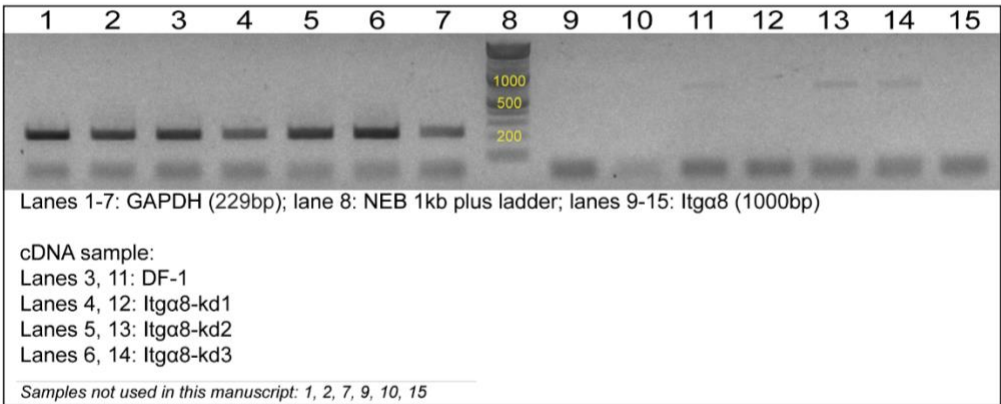

**Npnt-OE**

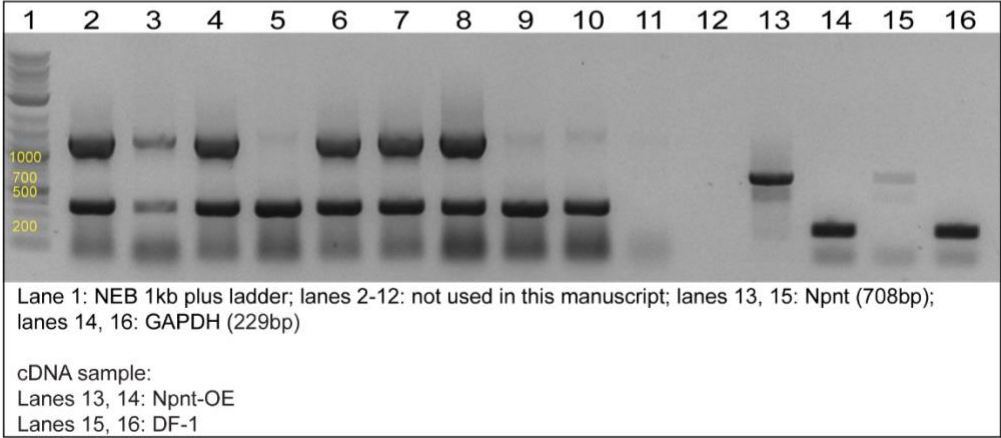

Supplement: Source data 2. [file elife-74307-data2.pdf]
